# Computational Modeling of Bubbles Growth Using the Coupled Level Set—Volume of Fluid Method

**DOI:** 10.3390/fluids5030120

**Published:** 2020-07-23

**Authors:** Amir Taqieddin, Yuxuan Liu, Akram N. Alshawabkeh, Michael R. Allshouse

**Affiliations:** 1 Department of Mechanical Science and Engineering, University of Illinois at Urbana-Champaign, Urbana, IL 61801, USA; 2 Department of Mechanical and Industrial Engineering, Northeastern University, Boston, MA 02115, USA; 3 Department of Civil and Environmental Engineering, Northeastern University, Boston, MA 02115, USA

**Keywords:** reactive flow, physicochemical hydrodynamics, s-CLSVOF, bubble growth and rising, transport of species, interfacial mass transfer

## Abstract

Understanding the generation, growth, and dynamics of bubbles as they absorb or release dissolved gas in reactive flows is crucial for optimizing the efficiency of electrochemically gas-evolving systems like alkaline water electrolysis or hydrogen production. To better model these bubbly flow systems, we use a coupled level set and volume of fluid approach integrated with a one-fluid transport of species model to study the dynamics of stationary and rising bubbles in reactive two-phase flows. To accomplish this, source terms are incorporated into the continuity and phase conservation equations to allow the bubble to grow or shrink as the species moves through the interface. Verification of the hydrodynamics of the solver for non-reactive systems demonstrates the requisite high fidelity interface capturing and mass conservation necessary to incorporate transport of species. In reactive systems where the species impacts the bubble volume, the model reproduces the theoretically predicted and experimentally measured diffusion-controlled growth rate (i.e., *R*(*t*) ∝ *t*^0.5^). The model is then applied to rising bubbles to demonstrate the impact of transport of species on both the bubble velocity and shape as well as the concentration field in its wake. This improved model enables the incorporation of electric fields and chemical reactions that are essential for studying the physicochemical hydrodynamics in multiphysics systems.

## 1. Introduction

Multiphase flows are encountered in many different scientific and industrial applications such as atomization in combustion [1], boiling in power generators [2], sedimentation flows [3], ocean waves [4], and electrochemically gas-evolving systems [5,6]. Understanding the dynamics of bubble-liquid multiphase flows is difficult due to the nonlinear, multiscale, and interconnected interactions between the two phases [7]. These systems become more complicated with flows that involve chemical reactions that result in reactive flows, such as bubbly flows in electrochemically gas-evolving systems that contain dissolved gas in the liquid phase. In these systems, the electrochemical reactions that occur at the electrode are responsible for the bubble formation, growth, and detachment at the electrode surface. The formed gas bubbles play a crucial role in controlling the performance of the electrochemical system. While the presence of bubbles enhances the system efficiency by inducing flow circulation due to the bubble rising within the cell, it has the unwanted effects of changing the conductivity of the bulk electrolyte and reducing the liquid-solid surface area of the electrode, which reduces the chemical reaction rates [8–11]. Efficient design of electrochemical systems, thus, requires accurately modeling bubble formation, growth, and flow, all of which are complicated by the growth and deformation of the bubble [12].

In order to model multiphase flows, several numerical methods have been developed to simulate two-phase flows and are generally classified as either one-fluid or two-fluid methods [13]. One-fluid methods, such as the level set (LS) [14], front tracking [15], and volume of fluid (VOF) [16] methods solve a single set of governing conservation equations encompassing both phases, in which the interfacial characteristics are determined by either capturing or tracking the interface between the two phases. Alternatively, the two-fluid frameworks, such as Eulerian-Lagrangian [17] and Euler-Euler [18] methods, evaluate the fluid dynamic fields by solving a set of conservation equations for each phase where the interphase interactions are approximated using correlation coefficients [13]. The one-fluid methods are more widely used compared to the two-fluid methods [13], since the one-fluid methods yield accurate results without using the coupling correlations necessary for the two-fluid methods [7,19]. The two-fluid methods are generally used to statistically model systems where bubbles have a high volume fraction or a large number of particles are present, and is typically used to investigate bulk flows on a larger scale. Studying fundamental bubble dynamics and capturing the transport of dissolved gas through the interface requires fine scale simulations to accurately capture the bubble interface. Thus, we adapt a one-fluid approach to investigate the fundamental aspects of bubbles in reactive flows.

One-fluid methods differ in terms of describing the interface between the two phases, which can be done either by using interface tracking or interface capturing techniques. The interface tracking methods track the interface with a high degree of precision; however, these techniques require more computational resources and are difficult to implement in cases where breakup or coalescence between interfaces occur [13]. Interface capturing techniques model the location of the interface by introducing a new field variable such as the volume fraction for the VOF method [20] or a signed difference function in the case of the LS method [21,22]. While no single method is perfect for application to model bubbles in reactive flows [13,23], the VOF and LS are widely used in simulating two-phase problems, particularly in simulating bubbles because of their efficiency [24–29]. Because the VOF method tends to smear the interface due to numerical diffusion, and the LS method can not guarantee conservation of mass, Albadawi et al., (2013) proposed coupling both LS and VOF approaches to model bubbles with accurate interface representation that conserve the bubble mass during the simulations [30]. The simple coupled LS and VOF (s-CLSVOF) method has been used in simulating bubbles in non-reactive flows in which the hydrodynamics are studied by solving the Navier-Stokes equations [30,31]. In reactive flows, modeling bubbles require coupling the Navier-Stokes equations with the transport of chemical species. Transport of species across the bubble interface controls the bubble volume growth or shrinkage based on the direction of the mass transfer fluxes. This work develops a one-fluid model that accounts for transport of species into bubbles that are allowed to grow or shrink as the species moves through the interface.

The change in the bubble volume, due to transport of chemical species, has a direct impact on the bubble hydrodynamics in the two-phase flow. These hydrodynamics, such as the buoyancy force, depend on the bubble volume. For example, a bubble growing in a reactive medium experiences higher buoyancy force as the volume increases, which will change the bubble dynamics such as the rising velocity. Studying the effects of material phase change due to the chemical species transport on bubble hydrodynamics requires coupling the fluid dynamic and transport of species physics. Several studies model bubbles rising with transport species without coupling both the fluid dynamics and transport of species together, keeping the bubble volume fixed [32–34]. Haroun et al., (2010) derived a model for transport of species incorporated into the VOF method to study the species transfer across the interface with applications to stable liquid films and structured packing [35]. Another model was developed by Marschall et al., (2012) to compute the mass transfer of species across a free interface using VOF method in OpenFOAM [36]. Both models provide similar results for the species transport problem [37]. It should be noted that these models did not consider the bubble interface changes due to the mass transfer. The complexity of tracking growing bubble interfaces is due to the coupling of mass transfer from transport of species into the continuity and other mass conservation equations such as volume fraction in VOF method.

In this paper, we present an algorithm and verification studies that couples the one-fluid transport of species model [35] with the s-CLSVOF method [30]. In Section 2, we discuss both the VOF and LS methods and how they are coupled by the s-CLSVOF method. The incorporation of the transport of species into the one-fluid approach is presented in Section 2.2. Section 3 focuses on validating the hydrodynamics of the model, and Section 4 presents results for how the bubbles are impacted by the incorporation of transport of species. Concluding remarks and further developments of the method are presented in Section 5.

## 2. Computational Model

To model the transfer of species through the bubble interface, the one-fluid approaches volume of fluid and level set methods are combined to provide the necessary high fidelity scheme to model the interface. These two approaches have previously been combined in the simple combined level set and volume of fluid (s-CLSVOF) to model the hydrodynamics of bubbly flow. The combined method is presented in Section 2.1. We present computational integration of the s-CLSVOF with the one-fluid transport of species in Section 2.2. Details on the implementation of this method are presented in Supplemental Materials A.

### 2.1. Simple Combined Level Set and Volume of Fluid Method

The failure to conserve mass by the LS method and inaccurate interface capturing in VOF methods prompted the development of another efficient technique to address these issues. Coupling these one-fluid computational methods benefits from the advantages of the two well-established methods and limit the disadvantages of using each of the single method separately. The coupling we implement, the simple CLSVOF method, combines the level set and volume of fluid methods [30,31,38]. The s-CLSVOF, method was developed by Albadawi et al. (2013) to study bubble behavior in non-reactive flows [30]. The coupled technique relies on the ability of LS to capture sharp interfaces and VOF to preserve mass. The s-CLSVOF method determines the interfacial properties (i.e., surface tension, contact angle, curvature) from a reinitialized LS interface and the fluid material properties based on the VOF’s volume fraction.

Simulating two-phase flows in the non-reactive case requires satisfying the mass and momentum equations. The mass conservation of each fluid is satisfied by imposing the continuity equation and the advection of the liquid volume fraction *α*. Momentum conservation is achieved by solving Navier-Stokes equation over the fluid domain. The equations are simplified by assuming the two-phase, immiscible fluid system is isothermal, and that both phases are incompressible with constant density. This assumption is reasonable for bubbles moving at a low Mach number and with low mass transfer rates.

Mass conservation and momentum conservation are given as follows:

(1) ∇⋅u=0,

(2) $$\frac{\partial (\rho u)}{\partial t}+\nabla \cdot (\rho uu)=-\nabla p+\nabla \cdot \tau +\rho g+f_{\sigma },$$

where ***u*** is the velocity field, *ρ* is the fluid density, *p* is the pressure, ***τ*** is the shear stress tensor that is defined as $\tau =\mu \left[(\nabla u)+{\left(\nabla u\right)}^{T}\right]$, *μ* is the fluid dynamic viscosity, the superscript *T* indicates the transpose of the vector, ***g*** is the gravitational acceleration, and ***f***_*σ*_ is the volumetric surface tension force. Computing ***f***_*σ*_ depends on the interface curvature and is calculated from the reinitialized LS interface. Since we are adapting a one-fluid approach, Equation (2) is solved only for a single effective fluid, but it is able to describe the two phases since the velocity of the effective fluid is given as the weighted average of the two phases as follows :

(3) $$u=(1-\alpha )u_{b}+\alpha u_{\ell },$$

where *α* is the volume fraction and the subscripts *b* and *ℓ* stand for the bubble and the liquid phases, respectively. Finally, the VOF method’s volume fraction advection equation

(4) $$\frac{\partial \alpha }{\partial t}+u\cdot \nabla \alpha =0,$$

ensures the conservation of the mass of each phase.

While the VOF method is used to advance the system through time, the LS method is used between steps forward in time to sharpen the interface. At the end of each time step, *α* is reinitialized using the LS method to sharpen the interface. The LS method removes the smearing inherent to the VOF while maintaining the total *α* and thus the mass of the phases is conserved. The more precise interface also helps in accurately calculating the curvature, normal vector, and surface tension force. At the conclusion of a given time step, the s-CLSVOF approach creates an initial LS function, *ψ*_0_, from the *α* field by the relationship

(5) $$\psi_{0}=(2\alpha -1)0.75\Delta x,$$

where *ψ*_0_ is the initialized LS function and Δ*x* is the computational cell width at the interface. Albadawi et al. (2013) selected this mapping as an approximation of the signed distance function where cells far from the interface are given a nominal value of ±1 and cells on or next to the interface get an intermediate value [30]. This initial mapping of the liquid volume fraction to the LS function must then be modified to sharpen the interface.

The reinitialization is carried out by solving the PDE

(6) $$\frac{\partial \psi }{\partial \tau }=sgn\left(\psi_{0}\right)(1-|\nabla \psi |),$$

where

(7) $$sgn\left(\psi_{0}\right)=\frac{\psi_{0}}{\sqrt{\psi_{0}^{2}+\Delta x^{2}}},$$

is based on the initialized level set function and *τ* is an artificial time not connected to the time marching of the VOF method. The Δ*x* is included in the denominator of the signed function to avoid indeterminate results [39].

A stopping condition has been determined, which yields a sharp interface without unnecessary extra iterations. Based on Equation (6), the level set function has converged when |**∇**_*ψ*_| = 1 [40]. Equation (6) is solved iteratively with Δ*τ* = 0.1Δ*x*. This provides a small enough artificial time to avoid large changes in the LS function till the stopping condition is reached. The number of needed iterations satisfies the condition:

(8) $$N_{corr}=\frac{1.5\Delta x}{\Delta \tau }.$$

where *N*_*corr*_ is an upper bound for the number of iterations [30]. For the given artificial time step, only a few iterations are generally needed. If the time steps in the VOF method are also small, the initialized value of *ψ*_0_ provides a good estimate of the converged level set, which also reduces the number of reinitialization iterations [30]. Once the level set function has converged, the relationship defined in Equation (5) is used to update *α*.

With the updated volume fraction field, it is necessary to also update the physical transport properties. The physical properties of the one-fluid (i.e., *ρ* and *μ*) are calculated by

(9) $$\rho =(1-\alpha )\rho_{b}+\alpha \rho_{\ell },$$

and

(10) $$\mu =(1-\alpha )\mu_{b}+\alpha \mu_{\ell }.$$

Finally, the surface tension force term, ***f***_*σ*_, in Equation (2) must be updated to account for the refined interface. The value of ***f***_*σ*_ is defined as

(11) $$f_{\sigma }=\sigma \kappa (\psi )\delta (\psi )\nabla \psi ,$$

where *σ* is the surface tension coefficient, *κ* is the interface curvature, which is defined as $\kappa =\nabla \cdot \hat{n}$, the normal vector of the interface is $\hat{n}=\nabla \psi /|\nabla \psi |$, and *δ* is the piece-wise function:

$$\delta =\{\begin{array}{ll} 0 & \text{if}\;|\psi |>1.5\Delta x,\\ \frac{1}{3\Delta x}\left(1+\cos\left(\frac{\pi \psi }{1.5\Delta x}\right)\right) & \text{if}\;|\psi |\leq 1.5\Delta x. \end{array}$$

The ranges in the *δ* function limit the surface tension force influence to a narrow region on or near the interface [30]. With these updated properties, the s-CLSVOF method can move forward in the time marching until completion.

### 2.2. Transport of Species Based on One-Fluid Approach

The s-CLSVOF method presented in Section 2.1 has been previously applied to systems with no interfacial mass transfer [30]. To incorporate the mass transfer that can occur in reactive systems, it is necessary to modify the governing equations and develop an algorithm to model the results. Here we present the modified governing equation.

In addition to the governing equations for the s-CLSVOF method, it is necessary to model the concentration transport of each chemical species, such as dissolved gas in the system. The species conservation is modeled based on one-fluid representation by the following diffusion-advection-reaction equation:

(12) $$\frac{\partial c_{i}}{\partial t}+\nabla \cdot \left(uc_{i}\right)=\nabla \cdot \left(D_{i}\nabla c_{i}-D_{i}\left(\frac{c_{i}\left(1-He_{i}\right)}{\alpha +He_{i}(1-\alpha )}\right)\nabla \alpha \right)+R_{i}.$$

where *i* is the chemical species index, *c* is the chemical species concentration per unit volume, *D* is the diffusion coefficient, *He* is Henry’s number, which is defined as the ratio of the concentration of species *i* inside the bubble over the concentration of the same outside the bubble, and *R* is the source term of the chemical reaction. Previous work has been done to incorporate this model of transport of species through into the VOF method [35,36], and this provides the foundation for our model.

The one-fluid representation of the species concentration relies on the liquid volume fraction:

(13) $$c_{i}=(1-\alpha )c_{b,i}+\alpha c_{\ell ,i},$$

and the diffusion coefficient is based on the harmonic mean

(14) $$D_{i}=\frac{D_{\ell ,i}D_{b,i}}{\alpha D_{\ell ,i}+(1-\alpha )D_{b,i}}.$$

Note that the harmonic mean of the diffusion coefficients reduces the spurious fluxes that can occur when there is a large difference in diffusivity between the phases.

The presented model of transport in Equation (12) is valid across the entire two-phase domain and satisfies the interface fluxes continuity and Henry’s law constraints. If the species being transported had a negligible effect on changing the size of the bubble, this equation in conjunction with the continuity and Navier-Stokes equations would be sufficient to model transport of species in bubbly flows [35,36].

The movement of concentration through the interface and the resulting phase change can impact both the local volume fraction and volumes of each phase if the species is highly concentrated or a material like dissolved gas. Accounting for this transport requires estimating the mass transfer of the concentration ${\dot{m}}_{i}$, which is linearly related to the concentration flux giving the relation as:

(15) $${\dot{m}}_{i}=MW_{i}\left(-D_{i}\nabla c_{i}+D_{i}\left(\frac{c_{i}\left(1-He_{i}\right)}{\alpha +He_{i}(1-\alpha )}\right)\nabla \alpha \right)\cdot \hat{n}.$$

where *MW*_*i*_ is the molecular weight of the species *i*. The reactive nature of the system is modeled by accounting for transport of species and phase change through the interface via source terms in Equations (1) and (4) to ensure mass conservation. It is also necessary to calculate the interfacial area *A*, which is defined as

(16) $$A=|\nabla \alpha |V_{c},$$

where *V*_*c*_ is the volume of the computational cell at the interface [41]. Note that |**∇***α*| = 0 inside the fluid and gas phases and |**∇***α*| ≠ 0 only at the interface.

To model the phase change resulting from the species passing through the interface, the volume fraction equation (Equation (4)) is modified to be

(17) $$\frac{\partial \alpha }{\partial t}+u\cdot \nabla \alpha ={\dot{\alpha }}_{s},$$

where ${\dot{\alpha }}_{s}$ is the volume fraction source term which represents the phase change. This source term is defined as

(18) $${\dot{\alpha }}_{s}=\frac{A}{V_{c}\rho_{\ell }}\sum_{i}{\dot{m}}_{i}.$$

Because *α* is defined as the liquid volume fraction, the mass transfer is divided by the density of the liquid to determine how much liquid has been displaced.

As the species passes through the interface, it changes phase, which changes the volume of the bubbles or the liquid. This is incorporated into the model by modifying the continuity equation (Equation (1)) to be

(19) $$\nabla \cdot u={\dot{v}}_{s},$$

where ${\dot{v}}_{s}$ is the source term due to the interfacial mass transfer. On a global scale, the source term is responsible for bubble growth or shrinkage within the reactive flow since it measures directly the change in the bubble volume. The source ${\dot{v}}_{s}$ links the fluid velocity and the mass transfer between the two-phases and can be expressed as:

(20) $${\dot{v}}_{s}=\frac{A}{V_{c}}\left(\frac{1}{\rho_{g}}-\frac{1}{\rho_{\ell }}\right)\sum_{i}{\dot{m}}_{i}.$$

The density difference in Equation (20) appears because the continuity constraint is applied for the two phases, which means this source term measures the transferred mass from one phase to the other.

## 3. Bubble Hydrodynamic Verification

In this section, we present several studies of rising bubbles in a viscous liquid first focusing on non-reactive systems to verify that the s-CLSVOF method properly captures the bubbly flow hydrodynamics. First, Section 3.1 presents two rising bubble case studies performed to determine the necessary grid resolution to ensure convergence. Then, the volume conservation of two and three-dimensional rising bubbles is verified in Section 3.2. Finally, in Section 3.3, we simulate bubbles rising in a viscous non-reactive liquid for a range of parameters and compare the obtained results with experimental and computational results from literature.

### 3.1. Grid Convergence Study

We run the convergence study using the s-CLSVOF solver by simulating a bubble rising in an initially quiescent viscous liquid in a two-dimensional domain without incorporating the transport of species modeling. Two bubble rising simulations are performed, which result in different shapes: Spherical and skirted bubbles. The height and width of the domain are, 18*R*_*o*_ and 6*R*_*o*_, respectively, with a structured uniform mesh. The no-slip side walls are used for the simulations and the top and bottom boundaries impose no normal gradients.

**Figure 1.**
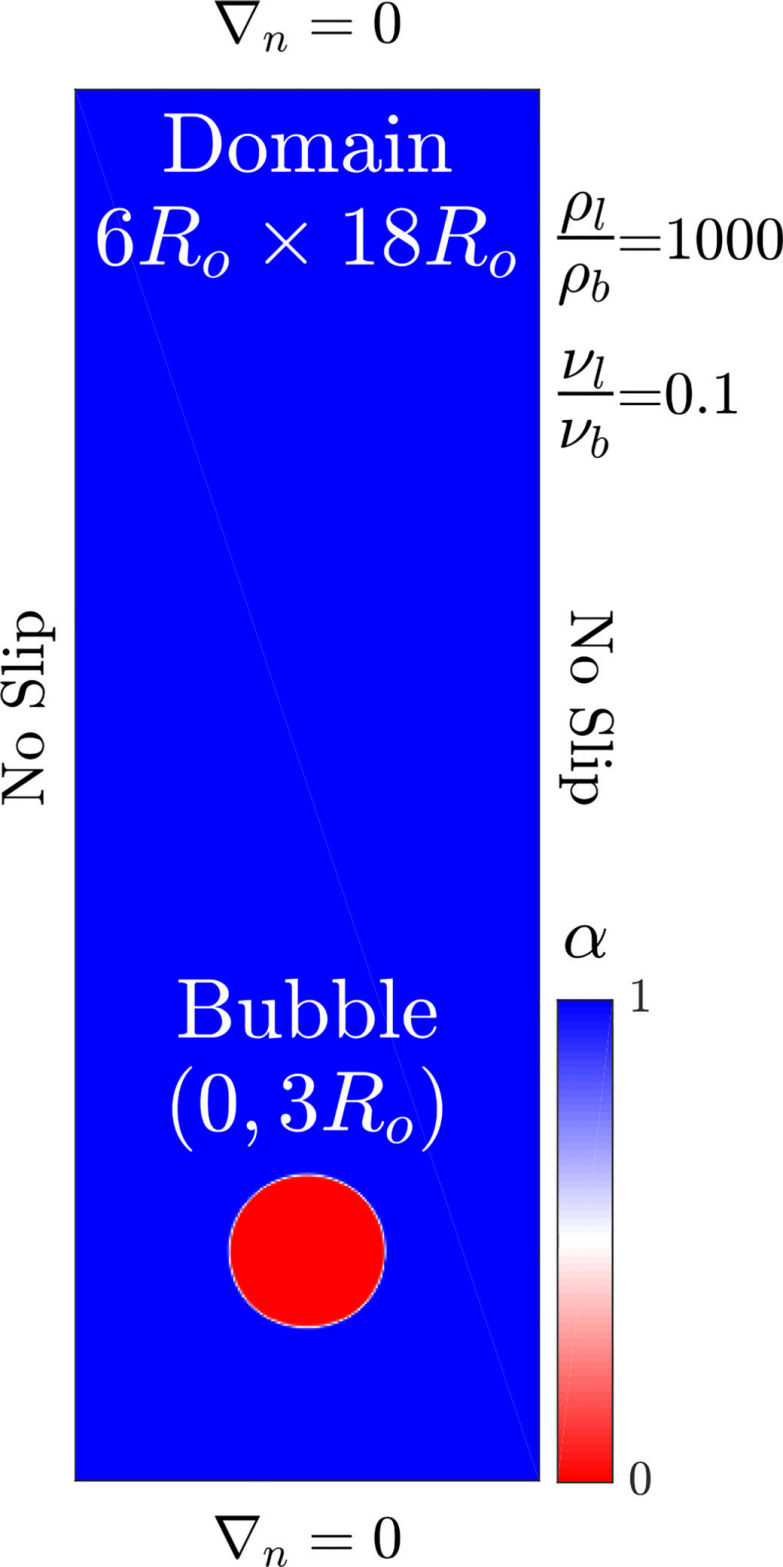
Computational setup of a bubble rising in an initially stagnant liquid. The initial liquid volume fraction is presented with the bubble (red) and liquid (blue).

The bubble starts freely rising from an initial position (0, 3*R*_*o*_) as shown in Figure 1. The initial bubble radius is *R*_*o*_ = 0.003 m for both simulations which is within the typical reported size range in the literature [30,38,42]. The density and viscosity ratios are set to 1000 and 100, respectively. To create the different bubble shapes, the spherical bubble has density and viscosity differences such that the Bond number is *Bo* = 17.7 and the Morton number is *M* = 711. The skirted bubble has the properties *Bo* = 243 and *M* = 266. Unlike our other simulations, which allow the time step to vary, a fixed time step is established by the ratio Δ*x*/Δ*t* = 1.2 for all simulations to ensure that the Courant number is approximately the same for all simulations.

**Figure 2.**
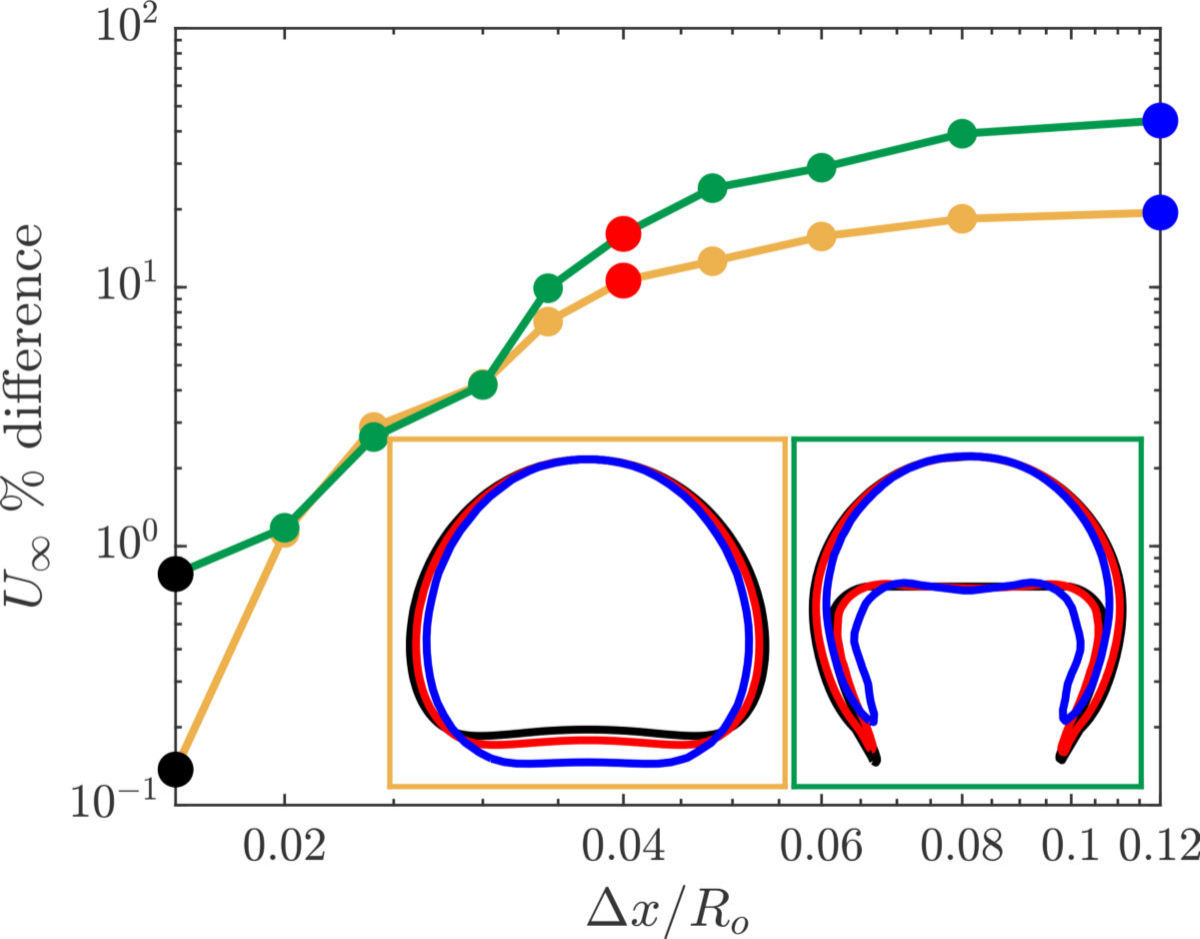
Mesh convergence study for a spherical and skirted rising bubble using the simple coupled level set (LS) and volume of fluid (VOF) (s-CLSVOF) solver in a non-reactive medium. The main figure presents the percent difference in the bubble terminal velocity with respect to the terminal velocity at Δ*x*/*R*_*o*_ = 0.0125 for a spherical (yellow) and skirted (green) bubble. The two insets at the bottom right present the bubble interface for different mesh resolution: Coarse mesh (Δ*x*/*R*_*o*_ = 0.12, blue), medium mesh (Δ*x*/*R*_*o*_ = 0.04, red), and fine mesh (Δ*x*/*R*_*o*_ = 0.016, black).

For the two bubble types, we simulated the rising bubble using eleven different resolutions with the coarsest mesh of Δ*x*/*R*_*o*_ = 0.12 and for the finest mesh of Δ*x*/*R*_*o*_ = 0.0125. The results of the rising bubble convergence study are presented in Figure 2. The figure shows the convergence of the terminal velocity for both the spherical and skirted bubble cases. The error percentage in Figure 2 corresponds to the difference in the bubble terminal velocity with respect to the terminal velocity for the finest grid size simulation.

For the coarse mesh, the skirted bubble has a larger error compared to the spherical bubble due to the bubble shape complexity, particularly the extended filaments at the bottom of the bubble. Note that the interface of the bubbles is always confined to within three cells, but for courser grids, this can correspond to a significant thickness relative to the bubble. For resolutions below Δ*x*/*R*_*o*_ < 0.03 there is less than 3% difference for both of the bubbles’ terminal velocity. At this degree of resolution, the interface is sufficiently sharp so that the shape of the bubble is well determined, and the interfacial forces are accurately calculated. The convergence study indicates that the s-CLSVOF simulations will be approximately mesh independent for grid cell sizes of Δ*x*/*R*_*o*_ ≈ 0.03 where the terminal velocity changes by less than 3% and the bubble shape remains relatively unchanged.

We also present the bubble shape at three different mesh resolutions in Figure 2. For both types of bubbles, the qualitative shape remains the same for the entire range of resolutions. In both cases, the shape does not significantly change between the resolution of Δ*x*/*R*_*o*_ = 0.04 (red) and Δ*x*/*R*_*o*_ = 0.016 (black). At the coarsest resolution, the bubble is slightly narrower. In the case of the spherical bubble, this results in an elongated bubble, and in the case of the skirted bubble, this produces thicker filaments. The thicker filament reflects the challenge of resolving the filament with relatively larger cells and demonstrates the importance of having a highly resolved interface for the more complex bubble shapes.

### 3.2. Volume Conservation of Rising Bubbles

Having established the necessary resolution to get converged terminal velocity and interface shape, we need to confirm that the s-CLSVOF method is mass conserving. To test the mass conservation, simulations of the spherical and skirted bubble rising from Section 3.1 are performed in both two and three-dimensional domains. In addition to the mass conservation results, this test provides an opportunity to confirm that the terminal velocity and bubble shape of the two-dimensional bubbles are similar to the three-dimensional bubbles given the same fluid properties. The fluid properties, bubble diameter, domain dimension, and boundary conditions are kept the same as the ones presented in Section 3.1. The mesh resolution for both domains is set to Δ*x*/*R*_*o*_ = 0.04 due to the computational cost of the three-dimensional domain. Unlike the previous section, which used a fixed time step, an adaptive time step is used for computational efficiency and convergence. The maximum Courant number and time step are *Co*_*max*_ = 0.25 and Δ*t*_*max*_ = 5(10^−4^) s, respectively.

**Figure 3.**
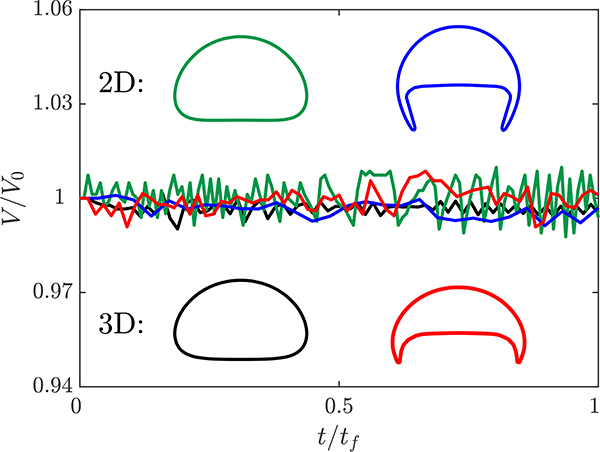
The bubble volume as a function of time normalized with respect to the final bubble volume. The normalized volume of the two (green) and three (black) dimensional spherical bubbles and the two (blue) and three (red) dimensional skirted bubbles are presented as a function of the normalized time. The shapes of the two (top) and three-dimensional (bottom) bubbles are presented as insets with the color of the interface corresponding to the normalized volume profile.

Figure 3 presents the normalized bubble volume as a function of time and the terminal shape of the bubble in both two and three dimensions. For all four simulations, the volume of the bubble changes by less than 1% throughout the entire simulation. It is valuable to note that there are no consistent trends to the error over the simulation indicating that there is no gradual growth or shrinking of the bubble. This indicates that s-CLSVOF is robust in terms of volume and mass conservation, which reflects the benefits of using the VOF method as part of the combined method.

For the spherical bubble simulations, the two and three-dimensional simulations produce almost identical bubble shapes. The results of the skirted bubble, however, produce different interface shapes for the two domains. Because the three-dimensional skirted bubble is wider, the projected area is smaller than the two-dimensional skirted bubble. Despite this difference, the shape itself remains a skirted bubble. For the spherical bubble, there is a less than 3% difference in the terminal velocity, but the more complex shape of the skirted bubble results in a 15% difference in the terminal velocity. It is possible that at higher resolutions the more complex skirted bubble terminal velocity will converge for the two domains, but due to the computational cost, we were not able to verify this. Overall though, the qualitative shape is accurately captured in two dimensions, and at least for the simpler bubble shape, the terminal velocity is accurately predicted by two-dimensional simulations.

### 3.3. Bubble Rising in Stagnant Liquid

**Figure 4.**
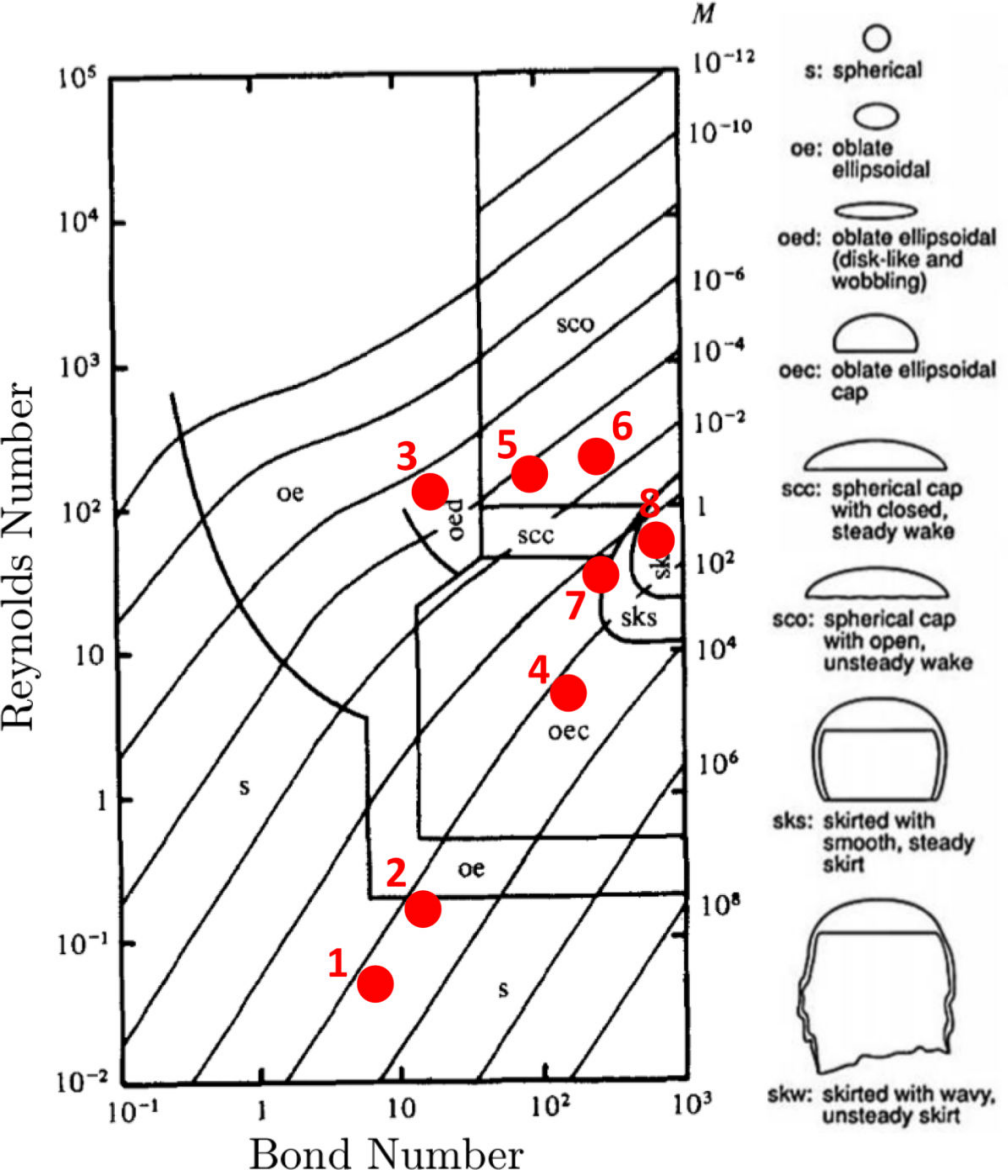
Bubble rising characteristic map based on the dimensionless numbers: Reynolds, Bond, and Morton numbers. The corresponding bubble shapes are depicted to the right. The red dots reference the simulations performed in the terminal velocity and shape verification study. This characterization map is reproduced with permission from Bhaga and Weber, 1981 [44].

Modeling of a single rising bubble in an initially stagnant liquid phase is a benchmark test case to verify the numerical simulations. Several experimental and numerical investigations have been devoted to bubbly flows focusing on the bubble’s terminal shape and velocity in viscous liquids [36,43–47]. The fluid characteristics of a single bubble rising are determined by the set of dimensionless numbers: Reynolds number $Re_{b}=U_{\infty }d_{b}/v_{\ell }$ representing the ratio of inertial and viscous effects, Bond number $Bo=g\left(\rho_{\ell }-\rho_{b}\right)d_{b}^{2}/\sigma$ representing the ratio of buoyancy and surface tension forces, and Morton number $M=g\left(\rho_{\ell }-\rho_{b}\right)\mu_{\ell }^{4}/\rho_{\ell }^{2}\sigma^{3}$ representing the ratio of viscous and surface tension forces. By setting two of these dimensionless numbers, the bubble shape can be predicted from the experimentally observed bubble flow map presented in Figure 4.

To validate the hydrodynamics of the model, we run two-dimensional simulations of a bubble rising in a quiescent viscous liquid without incorporating transport of species. The simulation domain, bubble size, and boundary conditions are set to match those presented in the Section 3.1. We perform eight different simulations where the bubble shapes vary based on setting the *Bo* and *M* values. These simulations are selected based on the experimental and numerical data to provide direct comparison between the s-CLSVOF method and a front-tracking method [44,46]. Two of these cases produce the spherical and skirted bubbles studied in Sections 3.1 and 3.2. The *Bo* and *M* are varied by changing the surface tension, fluid density difference, and fluid viscosity difference while keeping the ratio of these later two properties fixed. The *Bo* and *M* combinations simulated are presented in Figure 4. In all simulations, we use the structured uniform mesh with resolution Δ*x*/*R*_*o*_ = 0.024 and fixed time step Δ*t* = 5(10^−5^) s.

**Figure 5.**
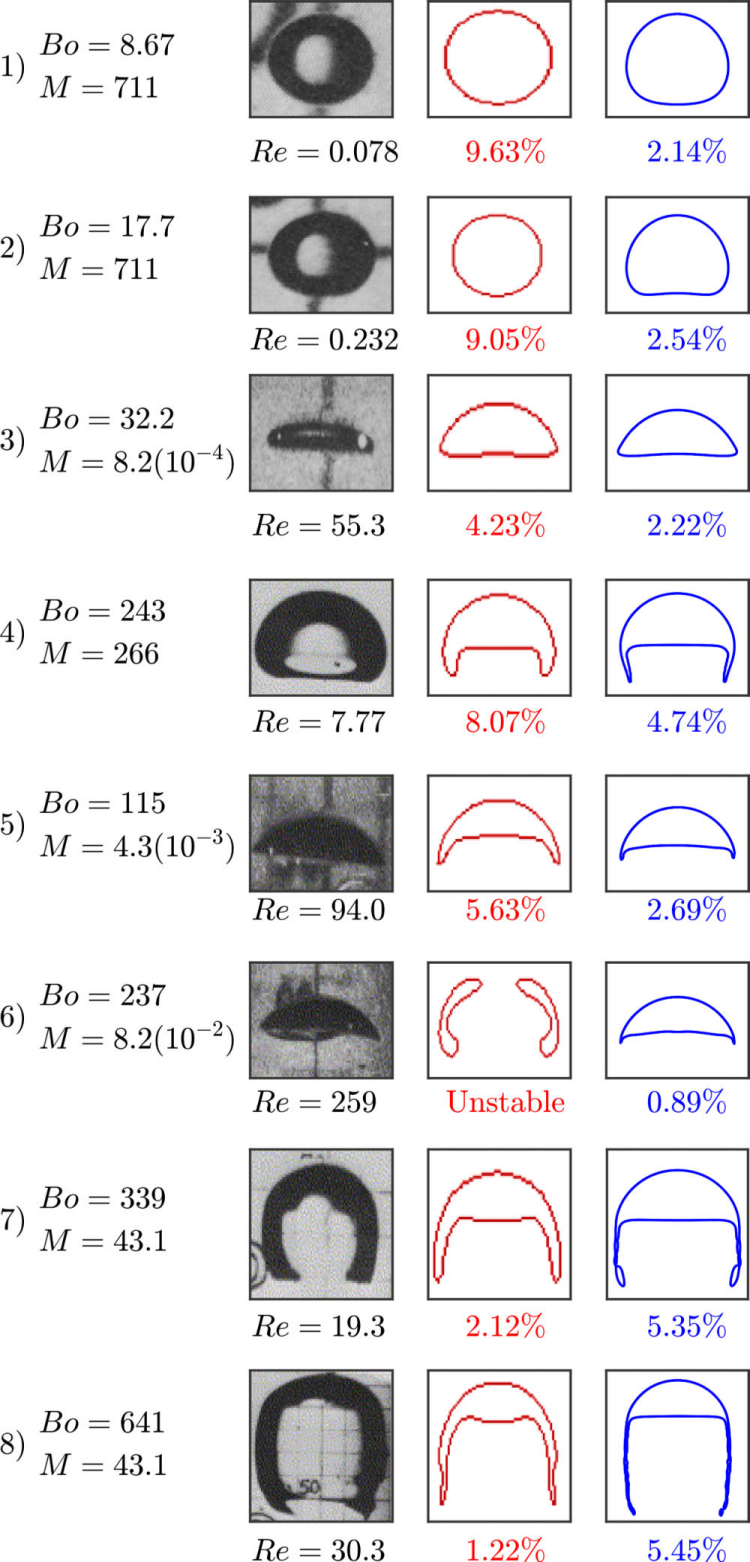
Bubble terminal shape and velocity in a non-reactive system: The experimental results are obtained by Bhaga and Weber, 1981 study [44], the front tracking results (red) are computed by Hua et al., 2007 using three-dimensional simulations [47], and the s-CLSVOF results (blue) are computed using two-dimensional simulations. The error percentage below the bubble figures are the absolute percent error between the experimentally measured and computed terminal velocity based on the *Re*.

The results of the rising bubble study are presented in Figure 5. We compare the bubble shape and terminal velocity to the experimental measurements by Bhaga and Weber, (1981) [44] and to the computational results by Hua et al., (2008) [46] who simulated rising bubbles using a front tracking method in a three-dimensional domain. Figure 5 presents the s-CLSVOF obtained terminal velocity percent errors, which are less than 5.5% compared to the experimental data. This is an improvement over the front tracking approach, which exceeds 9.5%. For cases 1–5, we obtained bubble shapes matching the experimental data and the front tracking results with a more accurate terminal velocity. In case 6, the s-CLSVOF method obtained a bubble shape that matches the experimental result with an accurate terminal velocity, which differs from the front-tracking approach, which was unable to get a stable bubble shape.

Cases 7 and 8 feature skirted bubbles where the simulation trends are slightly different. The experimental results of the bubble shape for cases 7 and 8 show the experimental bubble has a skirted shape with the filament pointing inward at the bottom, which is more closely captured by the s-CLSVOF simulations. While the s-CLSVOF results underestimate the terminal velocity by 5.35% and 5.24% for cases 7 and 8, respectively, the front tracking simulations produced lower error values for both cases. In the previous section, we identified some discrepancies between the two and three-dimensional skirted bubbles, which were possibly amplified by the requisite lower resolutions. For the comparison with experimental bubbles, a higher resolution is used which may be the reason for the reduced error, but there is still the potential that using two-dimensional simulations is causing the underestimation. Overall, the accurate modeling of the terminal velocity and bubble shapes of the s-CLSVOF method compared to experiments provide additional verification of the s-CLSVOF method to model bubbles rising in a non-reactive flow and improvements over existing models.

## 4. Impact of Transport of Species

Having examined the hydrodynamic accuracy of the method, we now incorporate transport of species into the simulations. Section 4.1 presents a bubble growth study in a stationary, reactive system and compares the results to theoretical growth rates. Then, the effect of transport of species on the velocity and shape of the rising bubbles are presented in Sections 4.2 and 4.3, respectively. Finally, we present the ramifications of incorporating transport of species on the concentration field left in the wake of a rising bubble in Section 4.4.

### 4.1. Bubble Growth Rate

In this section, we present a study where transport of species causes the bubble to grow but the bubble center of mass remains stationary. The results of these simulations can be compared to the well established behavior known as diffusion-controlled growth, in which the rate of species diffusion across the bubble interface controls its growth and is well known. The bubble radius in diffusion-controlled medium scales as *R*(*t*) = *Ct*^*β*^, where *C* is a constant that is determined by the concentration difference between the two phases and the associated diffusion coefficient, and *β* = 0.5 [48,49]. The bubble growth rate in our simulations can be directly compared to the experimentally verified bubble growth rate to confirm that the transport of species model is performing as expected.

The concentration difference between the bubble and the nearby liquid is not the sole factor that drives the transport of the concentration. As we showed in Section 2, the concentration flux is impacted by both the gradient of *c* and *He*. In fact, the *He* determines the steady-state concentration ratio between the inside and outside of the bubble. For the simulations in this section, *He* is greater than one, which requires the steady-state concentration inside the bubble to be larger than the concentration of the liquid to satisfy the *He* condition. Thus, the growth of the bubble takes place till the concentration inside the bubble becomes greater than the liquid concentration. In the case of these simulations, steady state is not achieved, so the experimentally observed growth rate is expected to persist after an initial transience.

The stationary flow simulation of single chemical species transport is carried out in a two-dimensional, square domain with edge length 32*R*_*o*_. The bubble is initially at the center of the domain with *R*_*o*_ = 0.005 m and zero concentration of the species inside the bubble. The concentration inside the liquid, *c*_∞_, is varied to ensure that there is limited variation in the bubble growth rate as a function of the concentration difference. The simulation properties used for this study are *g* = 0 to maintain the bubbles position, *D*_*ℓ*_/*D*_*g*_ = 1, *He* = 33, *ρ*_*ℓ*_/*ρ*_*g*_ = 100, *ν*_*ℓ*_/*ν*_*g*_ = 1, *MW* = 32(10^−3^) kg mol^−1^, and *σ* = 1 N m^−1^. The molecular weight and the Henry number correspond to dissolved oxygen in water at 25 °C. A structured uniform mesh with Δ*x*/*R*_*o*_ = 0.05 is used. All boundaries permit an outflow as the bubble grows and the concentration boundary condition is set to *c* = *c*_∞_ on all four boundaries.

**Figure 6.**
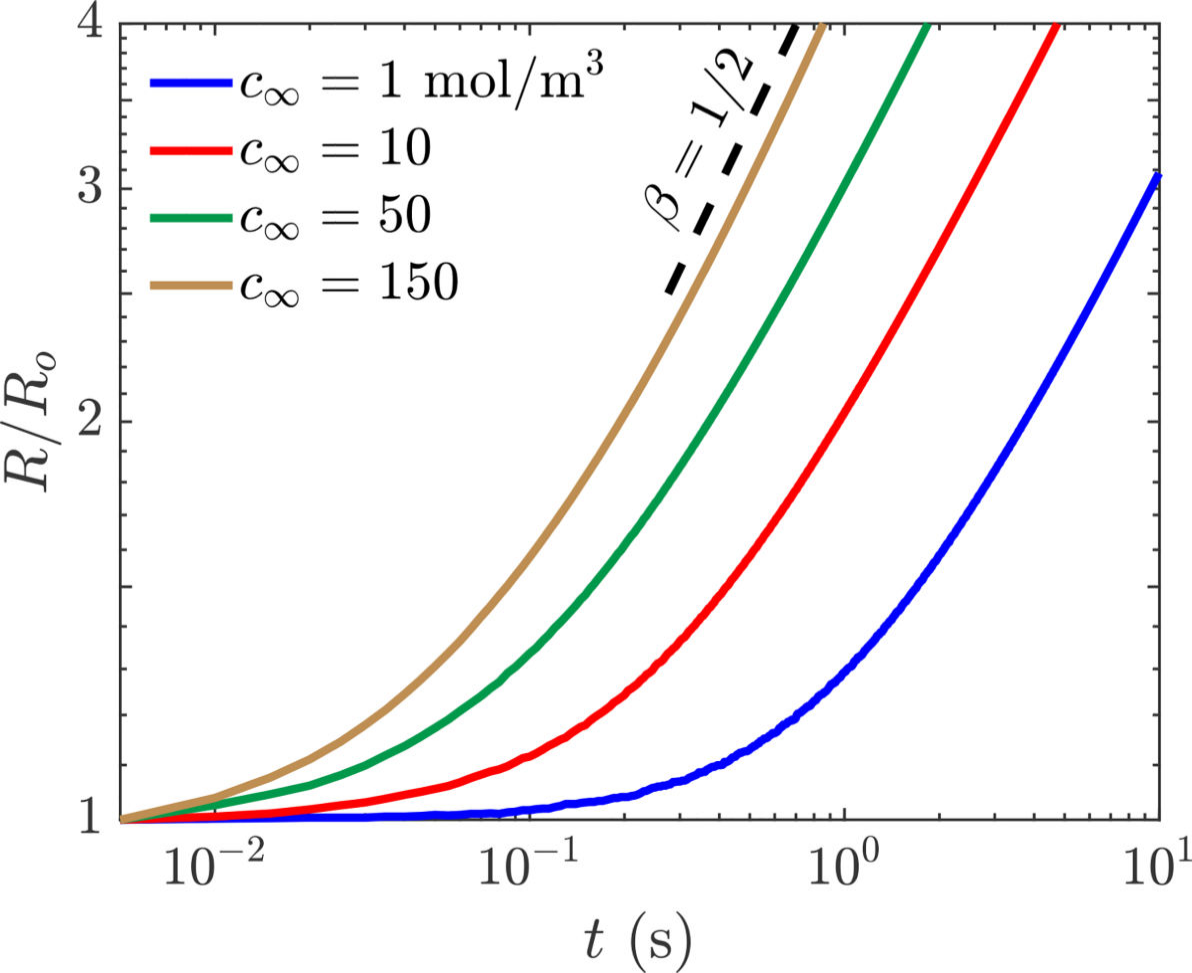
Simulation of bubble growth as a function of time with a liquid concentration *c*_∞_ of 1 (blue), 10 (red), 50 (green), and 150 mol m^−3^ (yellow). The obtained bubble radius as a function of time, *R*(*t*) *t*^*β*^, is normalized by the initial bubble radius. A power law fitting for *R*(*t*) ∼ *t*^0.5^ (black dashed) is presented for reference.

The bubble growth behavior as a function of liquid concentration is presented in Figure 6. The simulated growth curves reveal an initial transience then a steady-state growth that corresponds to the diffusion-controlled conditions. A fit has been calculated to determine the growth rate after the bubble has doubled in size for each of the concentration differences, and the growth rates *β* for each of the *c*_∞_ approach the theoretical 1/2. The power-law increase of the bubble radius with an exponent equals 1/2 is physically expected for homogeneous growth of stationary spherical bubble in a supersaturated solution where the volume fraction of the bubble is large [49]. As the liquid concentration *c*_∞_ increases, the bubble doubles in size, and the growth curve reaches the steady-state behavior faster. Because the growth rate coefficients closely match the experimentally observed and theoretically predicted growth rates, this indicates that the s-CLSVOF solver is accurately modeling the reactive interactions particularly for larger species concentration.

### 4.2. Velocity of a Rising Bubble in a Reactive Flow

Next, we simulate some of the bubbles from Section 3.3 to evaluate the effect of transport of species on the terminal velocity. The species modeled in the reactive flows are either volume conserving meaning that transport of this species does not impact the bubble volume, or non-volume conserving, which does change the bubble size as was the case in Section 4.1. Here, we run three combinations of species for each bubble case: One volume conserving species, one non-volume conserving species, and one of both types of species. Because the bubbles may be growing, terminal velocity is not necessarily achieved. We compare the velocity of the bubbles at the top of the domain to the terminal velocity of the bubble in the non-reactive flow. This comparison will allow us to relate the effect of the species transport on the bubble hydrodynamics in terms of bubble velocity.

The simulation properties are the same as those used to simulate the rising bubbles presented in Section 3.3. The initial concentrations inside the bubble and the liquid medium are equal to 0 and 1 mol m^−3^, respectively. The boundary conditions of the species are set to zero gradient at all boundaries, with *He*_*i*_ = 33, *MW*_*i*_ = 32 (g mol^−1^), and $D_{i}^{b}/D_{i}^{\ell }=1000$ for both species in all simulations. To ensure the transport of species is accurately simulated, the *Co*_*max*_ value is reduced to 0.25 to take smaller time steps.

**Figure 7.**
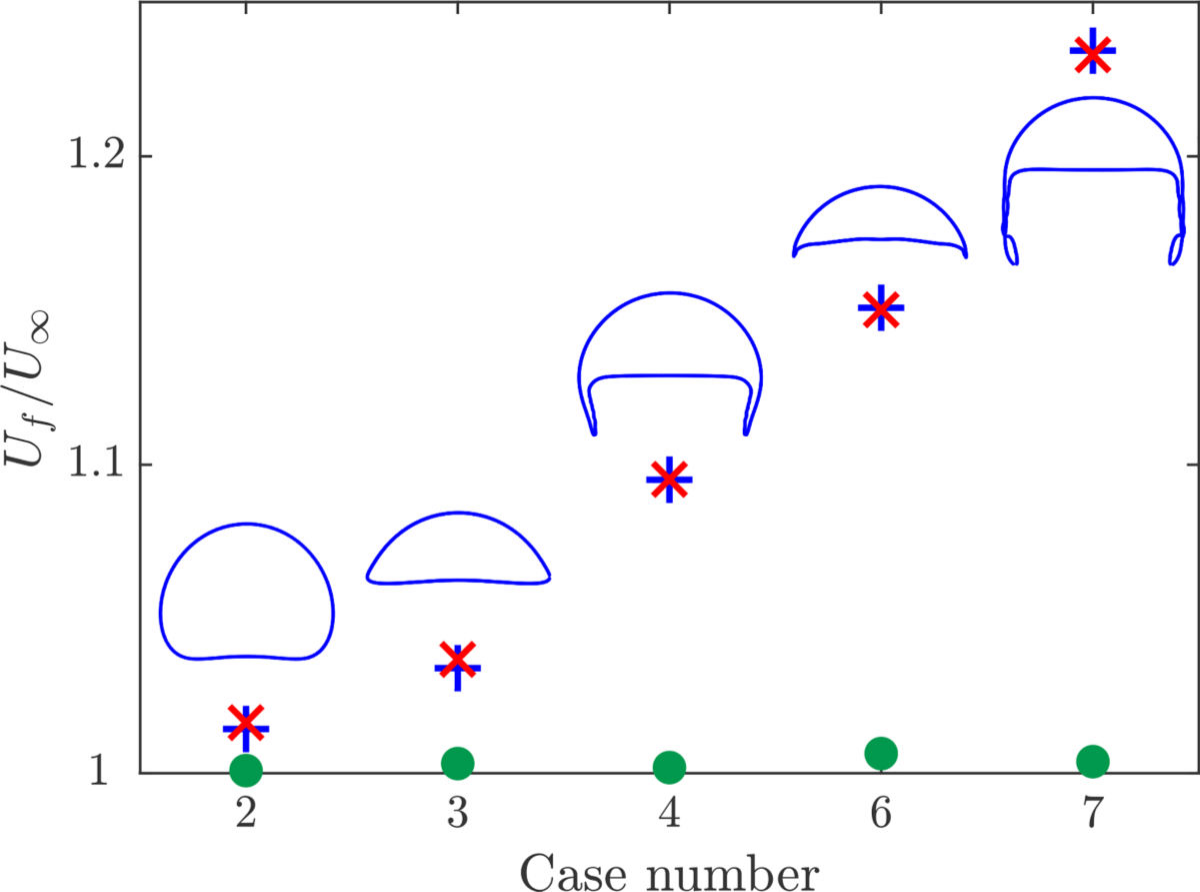
Comparison of the bubble velocity at the top of the domain *U*_*f*_ normalized by the non-reactive terminal velocity *U*_∞_ for simulations with one volume conserving species (green dot), one non-volume conserving species (blue cross), and both a volume and non-volume conserving species (red x). The steady-state bubble shape from the non-reactive simulation is presented for each case with the proper aspect ratio but not at the same scale.

The velocities of the rising bubbles normalized by the non-reactive terminal velocity are presented in Figure 7 where the case number corresponds to the case number in Figure 5. For the case where the species is volume conserving (green dots), the obtained velocity changes by less than 1%. This small discrepancy may be a result of the differences in the adaptive time stepping between the two simulations. When the species being transported impacts the bubble volume, all the bubbles grow and report higher velocities. The difference between the velocity increases as the perimeter of the bubble increases. The smallest increase in velocity is 3% for the spherical bubble, which has the smallest surface area. The largest increase in velocity is 24% for the skirted bubble, which has the largest surface area. Velocities increase due to the higher buoyancy force associated with the increased volume. Figure 7 also presents the maximum velocity for simulations where two species are included. In this case, only one of the two species increase the volume of the bubble and it is expected that the maximum velocity in this case will match the results when only the non-volume conserving species is present. This result is demonstrated in Figure 7 with a maximum difference in velocity of 0.3%. For all the cases tested, the bubble shape in the reactive cases remained the same as its non-reactive counterpart.

### 4.3. Shape of a Rising Bubble in a Reactive Flow

In the previous study, we demonstrated that including transport of species in the modelling of rising bubbles does impact the velocity of the bubble. In all cases, however, the shape of the bubble remained the same throughout the simulation. It is possible for bubbles to absorb enough dissolved gas for the shape of the bubble to change types relative to the non-reactive case. We perform a pair of studies here that demonstrate the potential for the bubble to change type as it grows or shrinks.

The simulations are similar to those performed in Section 4.2 except the initial radius for the bubble that will grow is *R*_*G*_ = 0.0025 m and the bubble that shrinks starts with a radius of *R*_*S*_ = 0.01 m. Because the shrinking bubble is larger than previous simulations the domain was expanded to be 6*R*_*S*_ × 9*R*_*S*_ with a mesh resolution of Δ*x*/*R*_*S*_ = 0.0072. The initial concentration of the dissolved gas in both the liquid and bubble and the boundary concentration are all 1 mol m^−3^.

**Figure 8.**
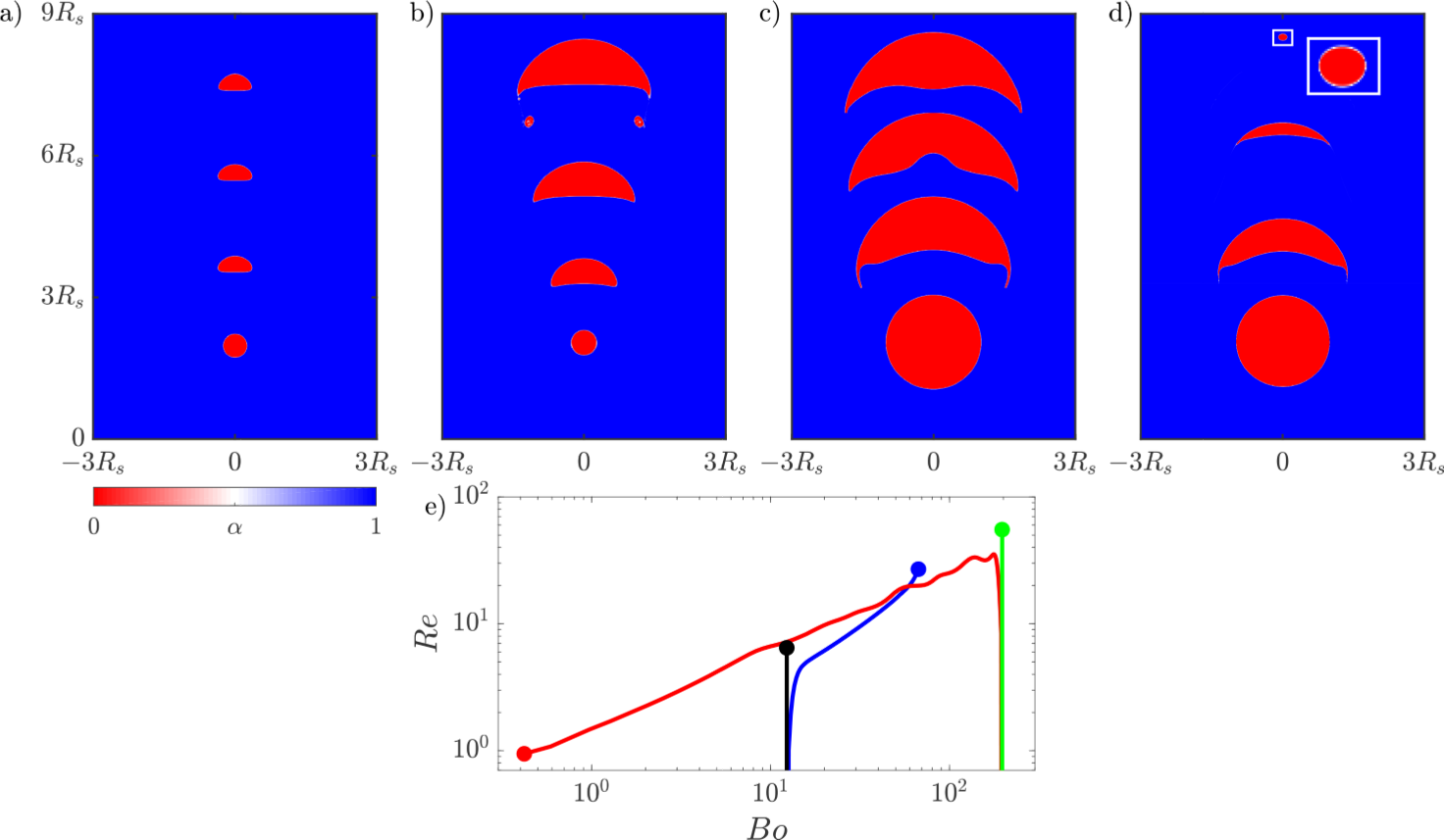
Evolution of an initially small rising bubble in (**a**) a non-reactive flow evolving into an ellipsoidal bubble and (**b**) the same initial bubble in a reactive flow growing into a skirted bubble. A larger rising bubble in (**c**) a non-reactive flow that becomes a skirted bubble and (**d**) the same initial bubble in a reactive flow shrinking to form a spherical bubble. Four time instances are shown for each bubble, and the dimensions of all four panels are based on the initial radius *R*_*s*_ of the larger bubble. An inset is included in (**d**) to enlarge the final state of the rising bubble. (**e**) The time evolution of the *Re* and *Bo* of the rising small bubble in non-reactive flow (black), small growing bubble (blue), large bubble in non-reactive flow (green), and large shrinking bubble (red).

We start by simulating a bubble rising in non-reactive flow with the initial flow parameters *Bo* = 12.3 and *Mo* = 0.10. The bubble position and shape at four time instances is presented in Figure 8a. The initially spherical bubble quickly deforms into the ellipsoidal shape. When a non-volume conserving species is included with *He* = 33, the bubble grows as it rises. The evolution of the growing bubble is presented in Figure 8b. By the time the bubble reaches the top of the domain, it is more than 11 times larger than its initial size. The shape of the growing bubble evolves from spherical, to ellipsoid, and finally to a skirted bubble that sheds smaller bubbles as can be seen at the final time instant in Figure 8b.

The second case considers a bubble that is initially four times as large as the bubble used for the growth case, and in this case, we investigate the impact of a non-volume conserving species escaping from the bubble. The initial flow parameters are *Bo* = 196.9 and *Mo* = 0.10. First the larger bubble is simulated without transport of species, and the evolution of the bubble is presented in Figure 8c. The initially spherical bubble quickly deforms into a skirted bubble shape, which stabilizes by the time it reaches the top of the domain. Then, the transport of species is included with *He* = 1/33, which will result in the bubble shrinking. The evolution of the shrinking bubble is presented in Figure 8d. The initially spherical bubble transitions from skirted to spherical as it rises. The final state of the bubble has been magnified in the inset to highlight the final shape of the bubble.

The transitions between shapes can be quantitatively observed when plotting the time dependence of the bubble’s *Bo* and *Re*, which both depend on the bubble volume. The change in these properties over time is presented in Figure 8e. The *Bo* and *Re* number are initially the same for the simulation of the smaller bubble in the non-reactive (black line) and reactive flows (blue line). After the growing bubble begins to absorb the species the two lines diverge and the *Bo* of the growing bubble begins to increase as the bubble accelerates upwards. The gradual increase in both the *Bo* and *Re* causes the growing bubble to leave the ellipsoidal bubble regime and ultimately enter the skirted bubble regime based on the bubble map in Figure 4. In the case of the larger bubble, the non-reactive (green line) and reactive (red line) bubbles have the same initial *Bo* and *Re* but quickly diverge. In this case, the shrinking bubble in the reactive flow decreases in volume so the *Bo* decreases, and as the bubble is rising, it is decelerating from its initial maximum velocity resulting in the *Re* decreasing. This decay in both the *Bo* and *Re* causes the bubble ultimately to move into the spherical region but not before reaching a skirted form at the begging of the simulation. In both the growing and shrinking cases, the bubble map gives a good indication of the instantaneous shape of the bubble despite this map being based on terminal bubble shapes.

### 4.4. Transport of Species Impact on Concentration Field

**Figure 9.**
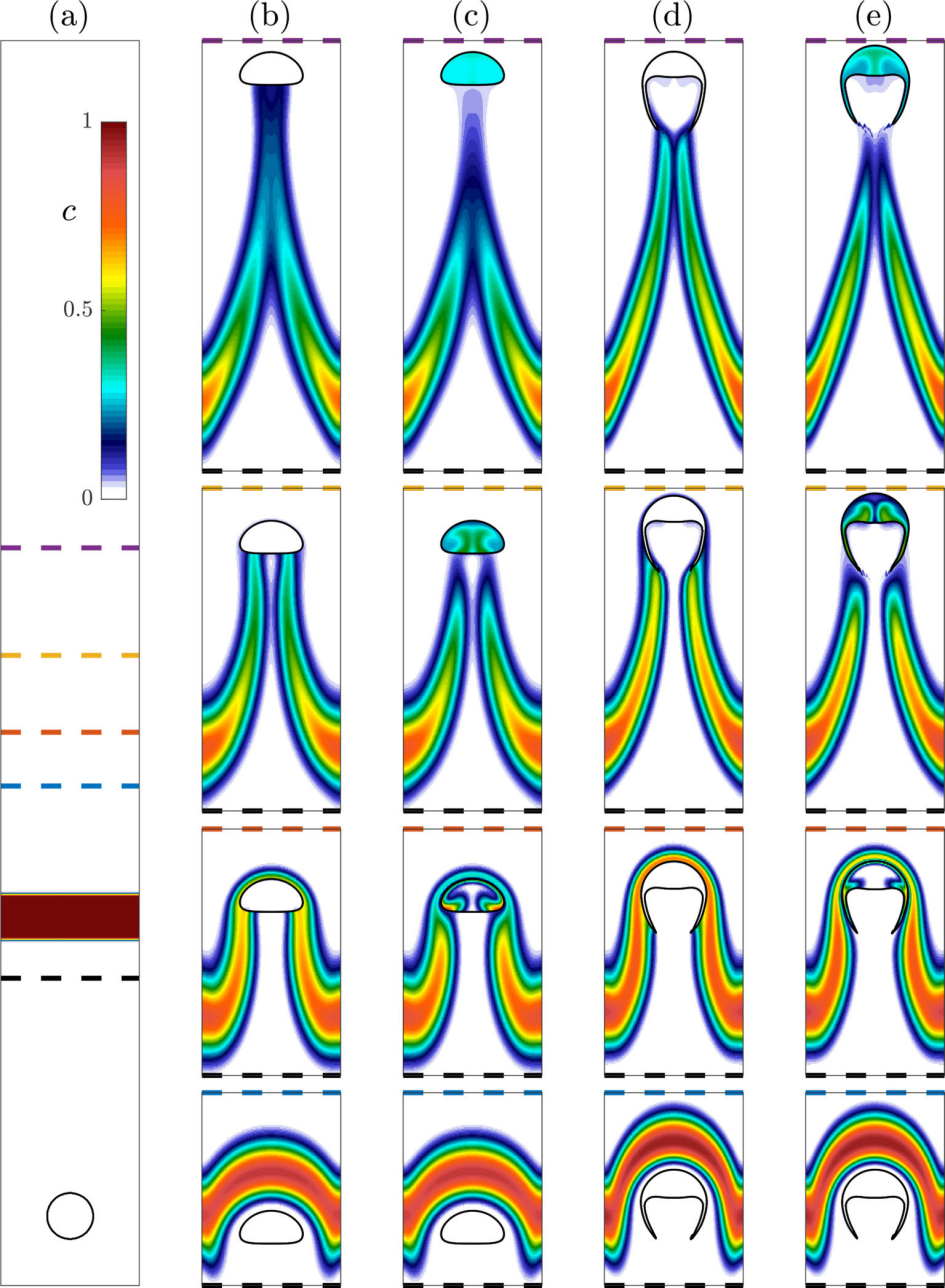
Evolution of four bubbles rising through a concentration layer. (**a**) The initial condition for all four simulations with a bubble containing *c* = 0 surrounded by liquid with *c* = 0 below a layer with *c* = 1. (**b**,**c**) Spherical and (**d**,**e**) skirted bubbles are allowed to rise through the concentration layer. Results for *He* = 0.01 are presented in (**b**,**d**) while *He* = 33 is presented in (**c**,**e**). Four different time instances with vertical ranges with the bottom of the range being the black dashed line and the top of each image corresponding to the blue, orange, yellow, and purple dashed lines in (**a**).

We have demonstrated how accounting for transport of species into the bubble will cause the bubble velocity and shape to change. Next, we demonstrate that the absorption of material into the rising bubble also impacts the concentration field in the liquid and cause a significant difference in the distribution of the concentration. To do this, four simulations were run with a rising bubble passing through a layer of high concentration. The bubble and surrounding liquid both initially have zero concentration as depicted in Figure 9a. This allows the bubble to rise and develop its steady state velocity and shape (spherical and skirted bubbles were studied) without transport through the interface. The bubble then passes through a region of high concentration where in two of the simulations *He* = 33 as in the previous sections, which allows concentration from the layer to be absorbed into the bubble. The other two simulations have *He* = 0.01, which effectively prevents transport from the layer into the bubble. Because the species is volume-conserving, the bubbles do not change shape.

The evolution of the concentration layer for the four simulations is presented in Figure 9b–e. As the bubble reaches the concentration layer, the layer initially deforms around the edge of the bubble as presented in the first (bottom) row. As the bubble moves through the concentration layer in the second row, transport is allowed in the *He* = 33 cases (c) and (e), but negligible transport is permitted in the *He* = 0.01 cases. After the bubble has passed through the layer, concentration fills the entirety of the *He* = 33 bubbles. At this point, the impact of the bubble absorbing concentration is apparent in the wake of the bubble. The concentration remains relatively high immediately behind the bubble in the *He* = 0.01 cases while it is depleted in the *He* = 33 case. This is further demonstrated in the last (top) row where the concentration in the *He* = 33 bubbles has become nearly uniform and there is limited concentration immediately below the bubble. Eventually the high concentration wake of the *He* = 0.01 case will detach from the bubble, and the impact of the bubble on the concentration field will stop. Because the *He* = 33 have absorbed a significant amount of the species, it will continue to slowly leak this concentration into the liquid as it rises. Ultimately this results in a greater impact on the concentration field.

## 5. Conclusions

Because multiphase flows and in particular electrochemical systems featuring chemical reactions are pervasive, there is a need to better model transport of species across the interfaces of the different phases in order to best optimize these systems. We have presented a one-fluid approach based on the s-CLSVOF method to simulate bubbles in systems where the transport of species has the potential to change the volume of the bubble. The s-CLSVOF method takes advantage of the sharp interfaces produced by the level set method and the volume conservation of the volume of fluid approach. The incorporation of source terms into the governing equations allowed us to expand the scope of the method to model bubbles that grow or shrink. The approach was implemented in OpenFOAM v4.1 and verification studies were performed to ensure that both the hydrodynamics and the transport of species are being faithfully modeled.

After describing the method and the algorithm, verification studies were presented to ensure the simulated bubble hydrodynamics matched results previously observed. A convergence study was performed on two-dimensional rising bubble simulations to determine the necessary grid resolution to achieve convergence for our system. Terminal velocities converged to less than 1% and a baseline for grid resolution was established. Mass conservation was next verified to within 1% for the two rising bubbles in both two and three-dimensional domains. Finally, the s-CLSVOF method was compared to experimental results [44] and front tracking simulations [46]. The eight cases used for comparison demonstrate that the s-CLSVOF method does an excellent job modeling the shape and terminal velocity of bubbles rising in a variety of shape regimes. The combination of these studies demonstrates the capacity of the method to accurately model the hydrodynamics of a bubble rising in a non-reactive system.

Having established the accuracy of the hydrodynamics, we next studied the impact of incorporating transport of species in the simulations. For a stationary bubble, we verified that the steady-state growth rate approximately follows the 1/2 power law predicted by theory and measured experimentally. Next, we incorporated transport of species into simulations of different types of rising bubbles. When the transported species did not impact the volume of the bubble, the simulations accurately reflect the negligible change in terminal velocity to less than 1%. When the species does impact the bubble volume, the maximum velocity increase by between 3% and 24% depending on the surface area of the rising bubble. Then, a pair of comparison studies were performed to demonstrate that the incorporation of transport of species can cause the rising bubble to change shape from the one predicted in the non-reactive case. For one of the studies, the bubble grows and transitions from an ellipsoid to a skirted bubble, and in the other, the bubble changes from skirted to spherical as it shrinks. Observing the time dependence of the *Bo* and *Re* numbers reflects the change in expected shape, which is a new measure that can link the bubble volume change to the bubble shape transitions. Finally, we demonstrated that modeling the transport of species better captures the impact of absorption of concentration and better represents the concentration field in the wake of the bubble.

The presented solver in this paper helps to simulate and understand the fundamental aspect of bubble hydrodynamics in reactive flows. While, this s-CLSVOF solver can now model physicochemical properties of stationary and rising bubbles in reactive flows, there are multiple avenues for further research. The presented solver has been tested with structured and stationary meshes, but the libraries used are capable of incorporating both an unstructured and adaptive mesh. By incorporating these meshes into the solver, it would be possible to more efficiently model the rising bubble as only the region near the interface must be highly resolved. The enhanced efficiency would then make it more feasible to extend the simulations to larger three-dimensional domains. Another important advancement would be to investigate how the presence of multiple rising bubbles in a reactive system impacts the global absorption or release of species. This could be valuable in optimizing the release of chemicals via bubbles, which has application in water remediation and better understanding oil spill propagation [50]. Finally, there are multiple physical processes that can now be built into the model. Of primary interest is the incorporation of both an electric field and chemical reactions. Solving Poisson’s equation in conjunction with the accurately modeled bubble would allow us to study the effects of applying an electrical field on the system of charged chemical species similar to the physics of electrochemical systems. With the accurate release or absorption of chemical species in the system, it would also be possible to model how bubbles could induce or inhibit chemical reactions in their wakes.

## Supplementary Material

Supplementary Material A
