# Supplementary material for: Computational Modeling of Bubbles Growth Using the Coupled Level Set—Volume of Fluid Method
Source: Eur J Mech B Fluids. Author manuscript; Available in PMC 2021 Aug 3. (PMC8329854; doi:10.3390/fluids5030120)
Supplement: Supplementary Material A [file NIHMS1643497-supplement-Supplementary_Material_A.pdf]

Supplementary Material A. Computational Modeling of Bubbles in Reactive Flows Using the Coupled Level Set - Volume of Fluid Method

In this supplementary material, we present a background of the volume of fluid (VOF) and level set (LS) computational methods in section (A1). We also discuss the implementation of the coupled level set (LS) - volume of fluid (VOF) method combined with transport of species in OpenFOAM in section (A2). Finally, section (A3) presents the improvement of s-CLSVOF over VOF methods through capturing the interfacial pressure of a stionary bubble compared to the theoretical Young-Laplace equation.

A1. Methods

The fundamentals of the VOF and LS methods are presented in sections (A1.1) and (A1.2), respectively.

A1.1. Volume of fluid method

One-fluid approaches, such as VOF, provide the computational ability to capture and model multiple fluid phases and the interfaces between them. Particularly, VOF is an Eulerian, one-fluid method, in which the interface is determined using a discrete scalar indicator function α that represents the volume fraction of one of the phases [1]. For two-phase systems like bubbly flow, the value of α in a given cell has a value of zero or one if the entire cell is entirely in the bubble or liquid phase, respectively. Cells where $0 < \alpha < 1$ contain both liquid and the bubble, which indicates that the interface passes through this cell. In an idealized system, the value of α will have intermediate values only where the interface exists; however, in numerical implementations of the volume of fluid method, the interface will "smear" meaning that there will be multiple layers of cells with intermediate values near the interface.

There are two dominant VOF approaches that differ in how they identify the interface between the two phases. The interface reconstruction approach uses different fitting methods to approximate the bubble interface, and the algebraic approach is the standard approach that uses only the scalar value to approximate the location of the interface as a set of cells [2]. The algebraic approach is used here due to its simple implementation compared to the interface reconstruction approach. In the algebraic schemes, the interface is presented by multiple cells with finite thickness [2]. These schemes of the VOF method model the interface via a scalar advection transport of the volume fraction α.

The volume fraction α is modeled as a continuous property that satisfies the scalar advection equation:

$$\frac{\partial \alpha}{\partial t} + \boldsymbol{u} \cdot \boldsymbol{\nabla} \alpha = 0, \tag{A1}$$

where $\boldsymbol{u}$ is the velocity field [3]. When initializing the problem, α is set to zero for cells with the cell center in the bubble and one for cells with its center in the liquid. During the methods time marching, the position of

Email address: `m.allshouse@northeastern.edu` (Michael R. Allshouse)

the interface is updated based on the advection transport of α. To capture an accurate and sharp interface, a smooth transition from α =0 to 1 is required. However, inherent to the VOF method is numerical diffusion, which causes the interface smearing, and because α is coupled with the mass and momentum conservation equations, this can cause errors in the interfacial forcing resulting in spurious velocities.

The VOF method has been used to study multiphase systems with different applications, such as bubbles [4], atomization [5], and water waves [6]. While the method is able to accurately conserve mass, better precision in locating the interface between the two phases is necessary, and several developments in VOF implementation have pursued this goal [7]. These developments include implementation of numerical schemes to ensure the value of α is properly bounded and improved efficiency so that higher resolution grids can be applied, which limits the impact of the smearing. Despite great effort, VOF still suffers from numerical diffusion of the interface, which reduces the accuracy of capturing interfacial properties, especially for interfaces that undergo large deformations such as bubble growth or shrinkage.

A1.2. Level set method

A precise and sharp interface can be achieved using the LS method. This method is also classified as an Eulerian one-fluid approach, but the method captures the interface between two phases using a signed distance function, ψ, called the level set function. If the interface is a closed curve Γ, the LS function has a value of zero on the interface. In the bubble, $\psi < 0$ and in the liquid $\psi > 0$ with the magnitude equal to the distance to the interface. The LS method models the interface by solving the evolution of the LS function using the scalar advection transport equation:

$$\frac{\partial \psi}{\partial t} + \boldsymbol{u} \cdot \hat{\boldsymbol{n}} |\boldsymbol{\nabla} \psi| = 0, \tag{A2}$$

where $\hat{\boldsymbol{n}}$ is the vector normal to the interface. The advection of the LS is similar to VOF advection; however, the LS interface is expressed by a sharp transition between the two different phases based on the sign of ψ. While, the LS method accurately produces sharp, sophisticated interfaces, this method is not guaranteed to conserve mass of the two phases because as time evolves ψ does not necessarily accurately maintain the signed distance property resulting in errors in the interface position [2].

To reduce these errors, reinitialization of the interface is possible, in which ψ is reinitialized during the time marching to maintain the signed function property [8]. The reinitialization concept is introduced to reduce the numerical errors due to the steeping and flattening effects in calculating the local gradients of ψ that are inherent to the method [9]. Section 2.1 presents the governing equations of the reinitialization, which can be done in between time steps to reconstruct the isocontours of ψ and then locate $\psi = 0$. Performing the reinitialization calculations can be carried out periodically or at every time step during the simulation, keeping in mind the additional computational cost [9].

The LS method is widely used in a variety of applications including multiphase flow [10], image processing [11], flame propagation [12], and biophysics [13] due to its simplicity. Despite the advantage of performing

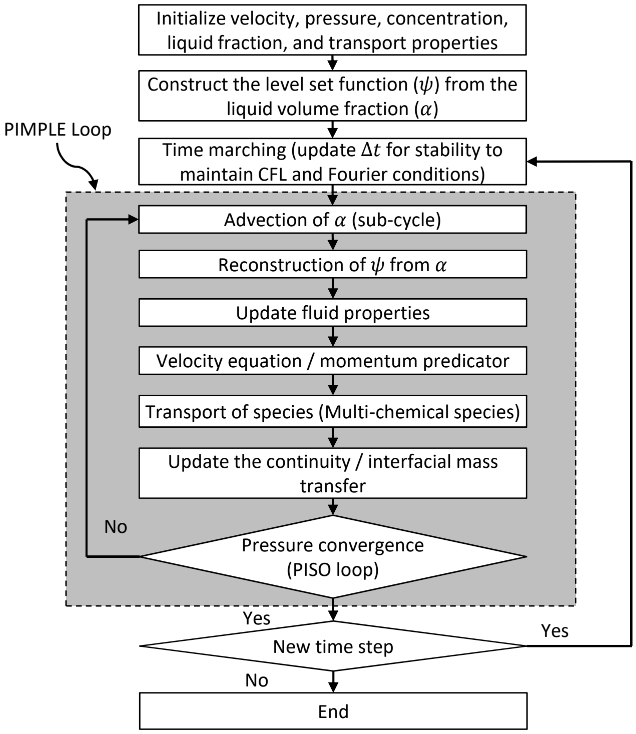

Figure A1: Schematic algorithm of the s-CLSVOF solver for reactive two-phase flows

interface reinitialization to enhance the numerical results in capturing $\psi = 0$, the mass conservation issue can still persist in LS implementations, especially for long time simulations.

A2. OpenFOAM implementation

To model a multiphase flow with transport of species, we implement the coupled solver s-CLSVOF in OpenFOAM v4.1 [14]. OpenFOAM is an open source CFD toolbox that primarily uses C++ to simulate fluid system. The existing packages provide a variety of solvers for many systems, and interFOAM is one package that can be used for multiphase problems [15]. This package, which was developed for VOF modeling of multiphase systems, provides a number of functions necessary for representing the interface and calculating the interfacial properties using the volume fraction, α. In this work, we use the finite volume PIMPLE algorithm to develop our multiphase s-CLSVOF bubbly flow method [14, 15]. The main advantage of the PIMPLE algorithm is the numerical stability for unsteady flow problems and the small number of corrector steps necessary to get converged time steps [14]. Within the explicit time marching loop, additional computational steps are performed to implement the coupling of the LS approach and transport of species. The proposed algorithm of the s-CLSVOF solver is presented in Fig. A1.

The algorithm begins by initializing the $\boldsymbol{u}$, p, c, and α fields based on the defined initial and boundary conditions of the particular problem. The initial one-fluid properties are determined based on the initial bubble position and the particular fluid and chemical species properties. Then, the algorithm uses Eq. (5) to calculate the initial level set function, ψ. Finally, the Courant-Friedrichs–Lewy (CFL) condition and Fourier

conditions are imposed to determine the first time step to be taken.

For the rising bubble simulations, an adaptive time step condition is used that will depend on the mesh resolution. The numerical stability is determined by the Courant number Co, which needs to satisfy the CFL condition:

$$Co = \sum_{i=1}^{n} \frac{u_{x_i} \Delta t}{\Delta x_i} \leq Co_{max}, \tag{A3}$$

where Δt is the fixed time step size, n is spatial dimension, u_{x_i} is the fluid velocity, Δx_i is the cell size, and Co_{max} is the maximum Courant number, which is $Co_{max} = 1$ for the non-reactive simulations in section 3 and $Co_{max} = 0.25$ for the reactive simulations in section 4. The lower Courant number is necessary to get converged result when transport of species is incorporated.

Following the time stability confirmation, the explicit PIMPLE loop starts by first advecting α with the velocity field. The advection of Eq. (17) is performed in OpenFOAM using the MULES technique, which is a flux corrected transport technique [15]. This technique imposes an additional limiter cutoff on the face-fluxes at the critical values to ensure the proper boundedness of the volume fraction independent of the numerical scheme, mesh resolution, or any unphysical parameters [15, 16]. After updating the α field, ψ is reinitialized using Eq. (5) and (6). The ψ field is then used to calculate all interfacial characteristics of the two phases and the normal vector of the bubble interface. The normal vector points outward from the bubble and the direction at a given computational face is expressed as follows:

$$\hat{\boldsymbol{n}}_{\boldsymbol{f}} = \frac{(\nabla\psi)_f}{|(\nabla\psi)_f + \epsilon|} \cdot \boldsymbol{S_f} \tag{A4}$$

where the subscript f stands for the computational face of the mesh cell, ϵ is a small (e.g. 10^{-10}) user-defined constant included to avoid division by zero, and $\boldsymbol{S_f}$ is the normal vector at the surface of the computational cell. The computed normal vector at the interface is used to evaluate the interface curvature and determine the surface tension forcing using Eq. (11). Then, the fluid and chemical species properties are updated throughout the domain using Eq. (9), (10), and (14).

Having calculated all the necessary properties and fields, we can begin solving the governing equations, so the algorithm moves to solve Eq. (2), and then Eq. (12). The computed concentration field is then used to evaluate $\dot{m}_i$ by Eq. (15). At this stage, we use the evaluated mass transfer and the computed interfacial area, A to calculate the volumetric source terms $\dot{\alpha}_s$ and $\dot{v}_s$ by solving Eq. (18) and Eq. (20), respectively. Finally, the later is used in solving the continuity constraint Eq. (19). Then, the pressure convergence condition is evaluated to determine if time marching can continue or if the algorithm must perform another iteration.

As part of modeling and solving the governing equations of the s-CLSVOF solver, we implemented some numerical changes to interFoam. The largest change is the incorporation of the level set reinitialization, but another important change is the inclusion of the source term in Eq. (19). This source impacts the momentum equation since the phase conservation and continuity equations are coupled. To satisfy this condition, OpenFOAM evaluates the momentum, Eq. (2), in a semi-discretized fashion in which Eq. (19) is

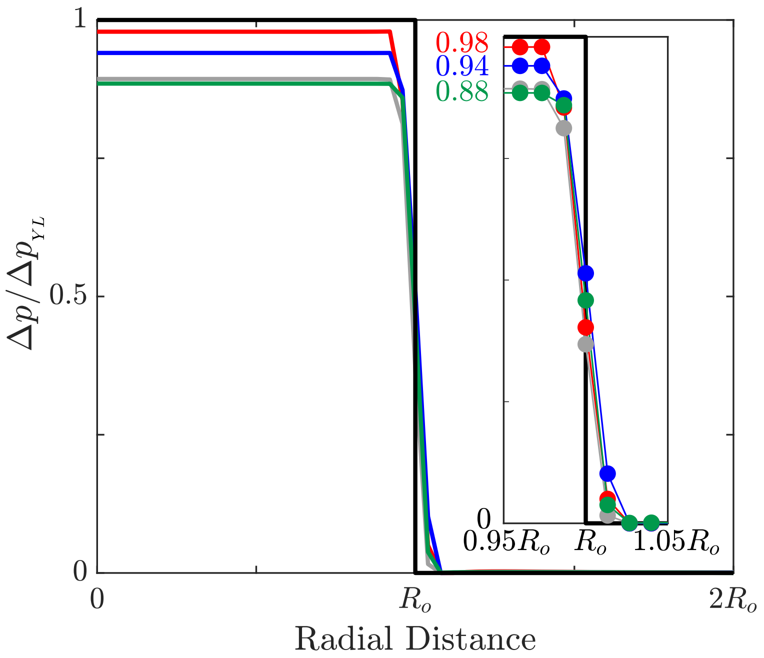

Figure A2: The interfacial pressure change of a stationary bubble inside a liquid domain normalized by the theoretical Young-Laplace equation (black line), for the two (blue) and three (red) dimensional s-CLSVOF simulation and the two (green) and three (gray) dimensional OF VOF simulation. The inset (right) focuses on the pressure change at the interface where each dot represents a cell center. In both cases, the x-axis shows the radial distance from the bubble center.

incorporated in the pressure equation, which is given in the form of the Poisson equation

$$\boldsymbol{\nabla} \cdot \left(\frac{1}{a_D} \boldsymbol{\nabla} p' \right) = \boldsymbol{\nabla} \cdot u - \dot{v}_s, \tag{A5}$$

where p' is the corrected pressure, a_D is the pressure matrix equation coefficients [17]. If the pressure convergence is achieved, the solver exits the PIMPLE loop and moves to the next time step till the simulation is complete. Otherwise, it returns to the volume of fraction advection to perform another iteration of the PIMPLE loop.

A3. Young-Laplace pressure

This initial study investigates a stagnant bubble in the fluid to demonstrate that the steady-state pressure difference across the bubble interface closely matches the theoretical Young-Laplace equation. This study serves to establish the improvement of s-CLSVOF in capturing the interface as compared to the VOF method. At the interface between the two fluid phases, the capillary pressure between two static fluids is described by the Young-Laplace equation, which relates the pressure jump to the curvature of the interface. For this equilibrium state, the pressure difference across the interface is $\Delta p = \sigma/R_o$ and $\Delta p = 2\sigma/R_o$ for two and three-dimensional bubbles, respectively, where Δp is the pressure difference across the interface, σ is the surface tension, and R_o is the bubble radius. It should be noted that for the Young-Laplace equation the curvature is $\boldsymbol{\nabla} \cdot \hat{\boldsymbol{n}}$.

For this study, we simulate a stationary bubble in both two and three-dimensional liquid domains using both the VOF and the s-CLSVOF methods with a bubble $R_o = 0.01$ m, $\sigma = 1$ N m^{-1}, square domain with edge length of $L = 4R_o$, and a uniform Cartesian mesh resolution of $\Delta x/R_o = 0.05$. The density and viscosity ratios between the two phases are 10 and 1, respectively, to match the conditions used in a previous applications of the VOF method [18]. The top and bottom boundaries have zero transverse velocity and zero normal gradient for pressure, velocity, and volume fraction. At the side walls, the boundary conditions for the velocity, pressure, and volume fraction are, respectively, no slip, zero flux, and zero normal gradient. The level set function has a to zero normal gradient boundary condition at all boundaries.

The resulting pressure profiles of the simulations are presented in Fig. A2. The pressure profile calculated by the s-CLSVOF method is closer to the theoretical prediction than the VOF method. Compared to the Young-Laplace pressure, the estimated error for the two and three-dimensional s-CLSVOF simulations are 6% and 2%, respectively, and the VOF simulations underestimate the pressure by 12% for both domains. The VOF results are close to the previously reported 9% error simulated with similar parameters [18].

The inset in Fig. A2 demonstrates that the interface thickness is three cells thick for both calculations though the s-CLSVOF supports larger pressure gradients. The interfaces are of similar thickness because the simulations are only for a small number of time steps, and the bubble is not deforming in any way. Greater smearing occurs for the rising bubble simulated using the VOF method. Despite similar thicknesses, the calculation of the LS function provides a more accurate approximation of the interface position and shape, which is the reason the s-CLSVOF method has a higher internal pressure. Additionally, the s-CLSVOF has reduced the presence of spurious velocities that are produced due to the numerical diffusion of α in the VOF simulation, which also contributes to the more accurate pressure in the s-CLSVOF simulations.

References

[1] C. Hirt, B. Nichols, Volume of fluid (VOF) method for the dynamics of free boundaries, Journal of Computational Physics 39 (1) (1981) 201 – 225. doi:10.1016/0021-9991(81)90145-5.

[2] C. R. Kharangate, I. Mudawar, Review of computational studies on boiling and condensation, International Journal of Heat and Mass Transfer 108 (2017) 1164 – 1196. doi:10.1016/j.ijheatmasstransfer.2016.12.065.

[3] J. Lopez, J. Hernandez, P. Gomez, F. Faura, A volume of fluid method based on multi-dimensional advection and spline interface reconstruction, Journal of Computational Physics 195 (2) (2004) 718 – 742. doi:10.1016/j.jcp.2003.10.030.

[4] A. Albadawi, D. Donoghue, A. Robinson, D. Murray, Y. Delauré, Influence of surface tension implementation in volume of fluid and coupled volume of fluid with level set methods for bubble growth and detachment, International Journal of Multiphase Flow 53 (2013) 11–28. doi:10.1016/j.ijmultiphaseflow.2013.01.005.

[5] L. Bravo, C. Ivey, D. Kim, S. Bose, High fidelity simulation of atomization in diesel engine sprays, Tech. rep., Army Research LAB Aberdeen Proving Ground MD Vehicle Technology Directorate (2015).

[6] M. M. Larmaei, T.-F. Mahdi, Simulation of shallow water waves using vof method, Journal of Hydro-environment Research 3 (4) (2010) 208 – 214. doi:10.1016/j.jher.2009.10.010.

[7] S. Mirjalili, S. S. Jain, M. Dodd, Interface-capturing methods for two-phase flows: An overview and recent developments, Annual Research Briefs (2017) 117–135.

[8] M. Sussman, P. Smereka, S. Osher, A level set approach for computing solutions to incompressible two-phase flow, Journal of Computational Physics 114 (1) (1994) 146 – 159. doi:10.1006/jcph.1994.1155.

[9] S. O. R. Fedkiw, S. Osher, Level set methods and dynamic implicit surfaces, Surfaces 44 (2002) 77. doi:10.1007/b98879.

[10] M. P. Kinzel, J. W. Lindau, R. F. Kunz, A multiphase level-set approach for all−−Mach numbers, Computers & Fluids 167 (2018) 1 – 16. doi:10.1016/j.compfluid.2018.02.026.

[11] X. Jiang, R. Zhang, S. Nie, Image segmentation based on level set method, Physics Procedia 33 (2012) 840 – 845, 2012 International Conference on Medical Physics and Biomedical Engineering (ICMPBE2012). doi:10.1016/j.phpro.2012.05.143.

[12] V. Mallet, D. Keyes, F. Fendell, Modeling wildland fire propagation with level set methods, Computers & Mathematics with Applications 57 (7) (2009) 1089 – 1101. doi:10.1016/j.camwa.2008.10.089.

[13] E. Maitre, T. Milcent, G.-H. Cottet, A. Raoult, Y. Usson, Applications of level set methods in computational biophysics, Mathematical and Computer Modelling 49 (11) (2009) 2161 – 2169, trends in Application of Mathematics to Medicine. doi:10.1016/j.mcm.2008.07.026.

[14] The OpenFOAM Foundation, http://www.openfoam.org/.

[15] S. M. Damián, Description and utilization of interfoam multiphase solver (2012).

[16] S. T. Zalesak, Fully multidimensional flux-corrected transport algorithms for fluids, Journal of Computational Physics 31 (3) (1979) 335 – 362. doi:10.1016/0021-9991(79)90051-2.

[17] M. Nabil, A. S. Rattner, interThermalPhaseChangeFoam—A framework for two-phase flow simulations with thermally driven phase change, SoftwareX 5 (2016) 216 – 226. doi:10.1016/j.softx.2016.10.002.

[18] N. Samkhaniani, A. Ajami, M. H. Kayhani, A. S. Dari, Direct numerical simulation of single bubble rising in viscous stagnant liquid, in: International Conference on Merchanical, Automobile and Robotics Engineering (ICMAR'2012), 2012.
